# 17-**β**-Estradiol Counteracts the Effects of High Frequency Electromagnetic Fields on Trophoblastic Connexins and Integrins

**DOI:** 10.1155/2013/280850

**Published:** 2013-05-30

**Authors:** Franco Cervellati, Giuseppe Valacchi, Laura Lunghi, Elena Fabbri, Paola Valbonesi, Roberto Marci, Carla Biondi, Fortunato Vesce

**Affiliations:** ^1^Department of Life Science and Biotechnology, Section of General Physiology, University of Ferrara, Via L. Borsari 46, 44121 Ferrara, Italy; ^2^Department of Food and Nutrition, Kyung Hee University, 1 Hoegi-dong, Dongdaemun-gu, Seoul 130-701, Republic of Korea; ^3^Interdepartmental Centre for Environmental Science Research, University of Bologna, Ravenna, Italy; ^4^Department of Biomedical Sciences and Advanced Therapy, Section of Obstetrics and Gynaecology, University of Ferrara, Ferrara, Italy

## Abstract

We investigated the effect of high-frequency electromagnetic fields (HF-EMFs) and 17-**β**-estradiol on connexins (Cxs), integrins (Ints), and estrogen receptor (ER) expression, as well as on ultrastructure of trophoblast-derived HTR-8/SVneo cells. HF-EMF, 17-**β**-estradiol, and their combination induced an increase of Cx40 and Cx43 mRNA expression. HF-EMF decreased Int alpha1 and **β**1 mRNA levels but enhanced Int alpha5 mRNA expression. All the Ints mRNA expressions were increased by 17-**β**-estradiol and exposure to both stimuli. ER-**β** mRNA was reduced by HF-EMF but augmented by 17-**β**-estradiol alone or with HF-EMF. ER-**β** immunofluorescence showed a cytoplasmic localization in sham and HF-EMF exposed cells which became nuclear after treatment with hormone or both stimuli. Electron microscopy evidenced a loss of cellular contact in exposed cells which appeared counteracted by 17-**β**-estradiol. We demonstrate that 17-**β**-estradiol modulates Cxs and Ints as well as ER-**β** expression induced by HF-EMF, suggesting an influence of both stimuli on trophoblast differentiation and migration.

## 1. Introduction 

Broadcasting systems and mobile phones generate high-frequency electromagnetic fields (HF-EMFs) ranging from 30 kHz to 300 GHz. As a consequence of their widely increasing diffusion human beings are today chronically exposed to such sources of energy, whose influences on physiological responses have not been yet exhaustively investigated. Data regarding the effects of these fields on human health are conflicting [1, 2]. Interestingly, *in vivo* human evaluation of brain glucose metabolism showed a significant increase upon acute cell phone radiofrequency signal exposure [3, 4].

As far as reproductive function is concerned, based on postnatal evaluation, no significant increase of reproductive risk was found in the rat following irradiation [5, 6]. However, a decrease in the number of mouse offspring, a prevalence of males over females, and an increase of stillbirth were also reported [7]. More recently it has been reported that the use of mobile phone decreases the human sperm count, motility, viability, and normal morphology [8, 9] probably due to oxidative stress [10]. 

During the first trimester of human pregnancy, extravillous trophoblast (EVT) cells invade the uterine spiral arteries generating a low-resistance, high-capacity uteroplacental circulation that ensures the success of gestation [11]. EVT cell functions are tightly regulated by multiple factors such as gap-junctional intercellular communication (GJIC) [12–14] and integrins [15, 16]. 

Gap junctions are membrane channels constituted by the association of two hemi-channels, termed connexons, each composed of six connexin (Cx) subunits. Gap junctions provide not only a pathway for the exchange of signaling molecules, but they are recognized as true signaling complexes regulating cell function and transformation [17]. Indeed, Cxs influence cell growth, development, and differentiation both in normal and pathologic conditions [17]. 

Integrins (Ints) are heterophilic cell adhesion molecules consisting of noncovalently connected *α* and *β* chains that together determine ligand-binding specificity and intracellular coupling. Thereby, Ints are the most important cell surface receptors for cell interactions with the extracellular matrix structures [18].

Changes in both Cxs and Ints [13, 16, 19, 20] expression have been reported during trophoblast differentiation to EVT.

Environmental stresses, including chemical pollutants [21], ionizing radiations [22], and oxidative stress [23], influence the expression of both Cxs and Ints. Moreover it is well documented that, in trophoblast tissue, the expression of these adhesion molecules is modulated by several hormones including estradiol [15, 24, 25]. This growth-promoting hormone affects placental function and embryo development both in primates and humans [26]. In addition it has been found to be involved in cytotrophoblast cell differentiation towards syncytiotrophoblast [26–29]. 

On the other hand, clinical effects of HF-EMF exposure on pregnancy are likely to occur, since it has been demonstrated that it results in increased levels of heat-shock protein 70 (HSP 70) in human amnion cells *in vitro* [30], although it does not influence the expression of this protein in human first-trimester extravillous-derived HTR-8/SVneo cells [31]. This cell line, derived from first-trimester human EVT, preserves all of their parental markers, as well as their responsiveness toward factors known to control EVT cell functions [32], thus representing a suitable model for the experimental study of early placentation process. In this experimental model we have shown that one hour exposure to GSM-217 Hz signals selectively modifies Cx mRNA expression pattern and protein localization [33]. 

The aim of the present work was to investigate whether HF-EMFs and 17-*β*-estradiol regulate cell-cell and cell-extracellular matrix interactions. To answer this question, we analyzed the effect of HF-EMFs, 17-*β*-estradiol, and their combination on both Cx and Int expressions inHTR-8/SVneo cells. Moreover, we studied the effect of HF-EMFs, 17-*β*-estradiol, and their combination on the estrogen receptor expression and immunofluorescence localization. Under the same experimental conditions, ultrastructural features were also evaluated.

## 2. Materials and Methods

### 2.1. Cell Cultures

The*  *HTR-8/SVneo cell line was kindly provided by Doctor CH Graham of Queen's University, Kingston, ON, Canada. Cells were grown in RPMI 1640 medium supplemented with 10% fetal bovine serum, 2 mM L-glutamine, 100 U/mL penicillin, and 100 *μ*g/mL streptomycin (Invitrogen Paisley, Scotland, UK). Cells were maintained at 37°C in normal atmosphere containing 5% CO_2_. For the experiments, cells were treated with trypsin, removed from culture flasks, and then seeded at a density of 1 × 10^6^ cells per 35 mmdiameter Petri dish. After 24–48 h culture, semiconfluent monolayers were exposed to treatments. 

### 2.2. Chorionic Villi

First-trimester human chorionic villi were obtained from consenting patients undergoing chorionic villous biopsy for prenatal diagnosis at the 11th week of gestation. Only tissues from physiological pregnancy were included in the study.

For RNA isolation, tissues were immediately frozen in liquid nitrogen and stored at −80°C.

### 2.3. Cell Viability Assay

Cell viability was assessed by MTT (3-(4,5 dimethylthiazol-2-yl)-2,5-diphenyl tetrazolium bromide; Sigma Chemical Co., St. Louis) assay in 0.01 M phosphate-buffered saline (pH 7.2). After 1 h cell incubation at 37°C, the formazan formed was extracted in 1 mL of DMSO for 1 h at 37°C and its absorbance measured at 510 nm in a spectrophotometer against a DMSO blank. The mean absorbance values obtained from 3 replicates were compared with controls. Inhibition of the MTT reduction potential of the HTR-8/SVneo cells was calculated and expressed as a percentage of control values following the method described [35], data not shown.

### 2.4. HF-EFM Exposure

All experiments consisted of control samples kept at 37°C and 5% CO_2_  in a Forma thermostat. Sham- and HF-EMF-exposed samples were kept in identical Forma thermostats which also housed the GSM-exposure system. Cells were exposed for 1 h to a 1.8 GHz sinusoidal wave, whose amplitude was modulated by rectangular pulses with a repetition frequency of 217 Hz [36] applied at time-averaged SAR values of 2 W/Kg.s, the safety limit for mobile phone emission according to INCIRP (International Commission on Non-Ionizing Radiation Protection). The exposure system was developed and built by the Foundation for Research and Information Technologies in Society (IT'IS Foundation, Zurich, Switzerland) following the specifications outlined in 5 [36] and extensively described in [37]. The system consisted of two 128.5 × 65 × 424 mm^3^ brass single-mode waveguide resonators operated inside the Forma thermostat. Each resonator was equipped with a plastic holder hosting six 35 mm Petri dishes arranged in two stacks. The carrier frequency, modulation, SAR level, and the periodicallyrepeated on and off exposure times were controlled by a computer. The exposure/sham conditions were assigned to the two waveguides by the computer-controlled signal unit. All exposure conditions and monitor data were encrypted in a file, which was decoded only after data analysis in order to ensure blind conditions for the experiment. Dosimetric field and temperature probes ensured that the temperature differences between sham (cells incubated into the waveguide resonator not selected for irradiation) and exposed cells at the standard condition of incubation was less than 0.1°C, ensuring no untoward thermal influence.

### 2.5. Estradiol Treatment

HTR-8/SVneo cells were treated for 24 h with 17-*β*-estradiol (Sigma Chemical Co., St. Louis, MO). Control and treated cells were maintained at 37°C by Forma thermostat in normal atmosphere containing 5% CO_2_ and stabilized in serum-free medium for 1 h before HF-EFM exposure. The optimal concentration of 10^−6 ^M 17-*β*-estradiol was chosen on the basis of a dose-response curve carried out in preliminary experiments (data not shown).

### 2.6. RT-qPCR (Reverse Transcription Quantitative Real-Time PCR)

Total RNA from 2 × 106 HTR-8/SVneo cells for each experimental condition was extracted with the AURUM total RNA Mini Kit with DNAse digestion (Bio-Rad, Laboratories, Inc., USA), according to the manufacturer's recommended procedure. After solubilization in RNAase-free water, total RNA was quantified by Bio-Rad SmartSpec Plus spectrophotometer (Bio-Rad, Laboratories, Inc., USA). First-strand cDNA was generated from 1 *μ*g of total RNA using iScript cDNA Synthesis Kit (Bio-Rad Laboratories, Inc., USA). As shown in Table 1, primer pairs were obtained from Primer Bank from the Real-Time PCR Primer and Probe Database, RT primerDB [34], to hybridise to unique regions of the appropriate gene sequence. The reverse transcriptase (RT-) PCR reactions were carried out using 1 *μ*L of cDNA in a 15 *μ*L total volume of PCR buffer (Invitrogen, Milan, Italy), containing 3 mM MgCl_2_, 300 *μ*M dNTPs, and 300 nM of appropriate primers. Taq polymerase (0.35 U) was also added. The amplification reactions were carried out in a thermal gradient cycler (Bio-Rad Laboratories, Inc., USA) for 40 cycles. Each cycle consisted of denaturation for 30 s at 94°C, annealing for 30 s at 60°C, and extension for 30 s at 72°C. A final extension step at 72°C for 5 min terminated the amplification. For each amplification, two types of controls were performed: (i) RT-PCR mixture with no reverse transcriptase to control for genomic DNA contamination and (ii) PCR mixture with no cDNA template, to check for possible external contamination. A 5 *μ*L sample of the PCR reaction was electrophoresed on an ethidium bromide-containing 2% agarose gel by the use of the Bio-Rad Subcell GT system.

Quantitative Real-Time PCR (qPCR) was performed using SYBR Green on iQ5 Multicolor Real-Time PCR Detection System (Bio-Rad Laboratories, Inc., USA). The final reaction mixture contained 1 *μ*L of cDNA, 300 nM of each primer, 7.5 *μ*L of iQ SYBR Green Supermix (Bio-Rad Laboratories, Inc., USA), and RNAse-free water to complete the reaction mixture volume to 15 *μ*L. All reactions were run as triplicates. The QPCR was performed with a hot-start denaturation step at 95°C for 3 min and then was carried out for 40 cycles at 95°C for 10 s and at 60°C for 20 s. The fluorescence was read during the reaction by the Opticon Monitor 3 software (Bio-Rad Laboratories, Inc., USA), allowing a continuous monitoring of the amount of PCR products. Primers have been initially used to generate a standard curve over a large dynamic range of starting cDNA quantity which allows to calculate the amplification efficiency (a critical value for the correct quantification of expression data) for each of the primer pairs. The melt curve analysis was performed at the end of each experiment to verify that a single product for primer pair was amplified (data not shown). As to control experiments, gel electrophoresis was also performed to verify the sizes of the amplified QPCR products. Ribosomal protein L13a (RPL13a), L11a (RPL11a), and GAPDH were used in our experiments as internal standards. As previously described, samples were compared using the relative cycle threshold (CT) method [38]. The fold increase or decrease was determined relative to a control after normalising to RPL13a (internal standard). The formula  2^−ΔΔCT^  was used, where ΔCT is (gene of interest CT) − (RPL13A CT), and ΔΔCT is (ΔCT experimental) − (ΔCT control). 

All the primers for qPCR analyses were GenBank obtained from Invitrogen (Invitrogen, Carlsbad, CA), and the sequences are listed Table 1.

### 2.7. Western Blot

After the experimental treatments, cells were washed with ice-cold phosphate-buffered saline solution (PBS), detached by scraping and transferred to eppendorf tubes. After 10 min centrifugation at 800 ×g at 4°C, the pellet was resuspended in ice-cold 10 mM Na-phosphate buffer, pH 7.4, containing 1% Nonidet-P40, 0.5% Na deoxycholate, 0.1% SDS, 1 *μ*g/mL of pepstatin A, E-64, bestatin, leupeptin and aprotinin, and 25 *μ*g/mL of PMSF. After 30 min on ice samples were centrifuged at 9,000 ×g at 4°C for 20 min. The supernatant was diluted 1.5 times with Laemmli buffer, boiled for 5 min, and kept at –20°C until use. Sample proteins were assessed according to Lowry et al. [39] using bovine serum albumin as standard. Western blotting procedures were carried out as we previously reported [29]; briefly, electrophoresis was carried out with a Mini Protean III apparatus (28 mA, 2 hours at 4°C), and the resolved proteins were transferred onto a nitrocellulose membrane (300 mA, 1 h at 4°C). Connexin, integrin, and estrogen receptor proteins were assessed by using Cx40, Cx43, Cx45, Int *α*1, Int *α*5, Int *β*1, and ER-*β* rabbit polyclonal antibodies (Santa Cruz Biotechnology Inc., CA, USA) against Cx, integrin and estrogen receptor *β* proteins were assessed by using rabbit polyclonal antibodies (Santa Cruz Biotechnology Inc. CA, USA) against proteins of human origin as primary antibodies (1 : 200). All antibodies were incubated overnight and, after washings, with goat anti-rabbit IgG colorimetric kit (Invitrogen, Carlsbad, CA) for 1 h. Immunoblots were developed by enhanced colorimetric reagent kit (Invitrogen, Carlsbad, CA), and a densitometric analysis of the band intensities was performed by the Gel Doc 2000 video image system (Bio-Rad Laboratories, Hercules, USA). Actin polyclonal antibody (Santa Cruz Biotechnology Inc., CA, USA) was used as an endogenous control for normalization. Values within each experiment were normalized to the control sample.

### 2.8. Indirect Immunofluorescence Staining of ER*β*

HTR-8/SVneo cells on coverslips were stained using rabbit polyclonal antibodies (Santa Cruz Biotechnology) raised against the human ER*β* protein (H-150, working dilutions 1 : 200 in PBS containing 0.05% BSA and 0.1% sodium azide). Cells were incubated with the primary antibodies for 1 h at room temperature (RT) and with secondary FITC-labelled goat anti-rabbit IgG serum (Santa Cruz Biotechnology) diluted 1 : 100 in PBS, for 1 h at RT in the dark. Slides were mounted in Vectashield (Vector Laboratories, Burlingame, CA, USA) antifading and examined using an Epifluorescence microscope (Nikon Eclipse E800; Nikon Corporation, Surrey, UK) equipped with a plan apochromat 100 × 0.5–1.3 oil immersion objective and a mercury lamp source. Amplifier and detector optimising parameters were maintained constant for all the experiments.

### 2.9. Ultrastructural Study

Cells were scraped and collected in 0.1 M cacodylate buffer (pH 7.4) and then spun in 1.5 mL tubes at 2,000 ×g for 5 min. Pellets were fixed with 2.5% glutaraldehyde in 0.1 M sodium cacodylate buffer for 4 h at 4°C. They were then washed with 0.1 M cacodylate buffer (pH 7.4) three times and postfixed in 1% osmium tetroxide and 0.1 M cacodylate buffer at pH 7.4 for 1 h at room temperature. The specimens were dehydrated in graded concentrations of ethanol and embedded in epoxide resin (Agar Scientific, 66A Cambridge Road, Stansted Essex, CM24 8DA, UK). 

Cells were then transferred to latex modules filled with resin and subsequently thermally cured at 60°C for 48 h.

Semithin sections (0.5−1 *μ*m thickness) were cut using an ultramicrotome (Reichard Ultracut S, Austria) stained with toluidine blue, and blocks were selected for thinning. Ultrathin sections of about 40–60 nm were cut and mounted onto formvar-coated copper grids. These were then double-stained with 1% uranyl acetate and 0.1% lead citrate for 30 min each and examined under a transmission electron microscope, Hitachi H-800 (Tokyo, Japan), at an accelerating voltage of 100 KV.

### 2.10. Statistical Analysis

All data were subjected to statistical analysis using PRISM software (version 2.1, Graph Pad Inc.). Data were examined by Bonferroni's posttest (2-way ANOVA). Specifically, tests were performed to ensure that the sham-exposed samples were not significantly different from one another. When these conditions were met, a second 1-way ANOVA was performed on the data from the sham and HF-EMF exposed groups. In all cases statistically significant difference was accepted when *P* < 0.05.

## 3. Results

The aim of this work was to investigate the effects of electromagnetic fields on cell-cell and cell-matrix interactions in the first trimester of pregnancy and the control of these effects by the 17-*β*-estradiol. To answer this question, we analyzed the effect of HF-EMF, 17-*β*-estradiol, and their combination on the expression of Cx and integrins in HTR-8/SVneo cells.

### 3.1. Viability of HTR-8/SVneo Cells

As revealed by MTT test, there was no significant difference in cells viability between the negative control (incubator) and the sham-exposed cells. Viability of exposed samples (1 h to GSM-217 Hz signal) was always greater than 98% with respect to sham-exposed samples data not shown.

### 3.2. Effect of HF-EMF, 17-*β*-Estradiol, and Their Combination on Cx Expression in HTR-8/SVneo Cells

**Figure 1:**
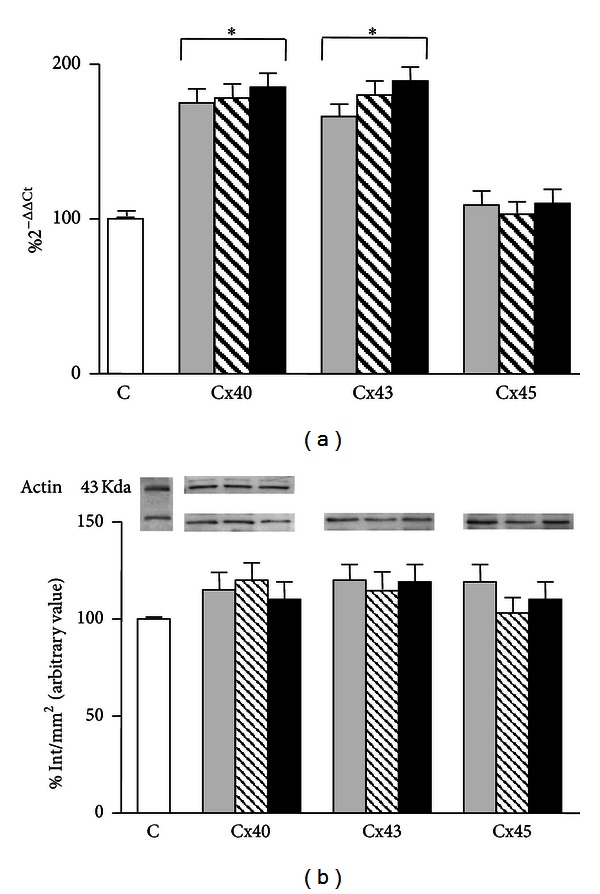
(a) Effect of HF-EMF (grey bar), 10^−6 ^M 17-*β*-estradiol (hatched bar), and their combination (black bar) on the expression of Cx40, Cx43, and Cx45 mRNA in HTR-8/SVneo cells. Results are expressed in %  2^−ΔΔCT^ with respect to the control value (white bar). (b) Western blot detection of CXs protein expression in HTR-8/SVneo cells HF-EMF exposed. Representative immunoblots of Cx40, Cx43, and Cx45 are shown. Results are the means ± SEM of three independent experiments, each analysed in triplicate. Data are means ± SEM of at least three independent experiments. *P < 0.001 versus sham-exposed cells (one-way ANOVA followed by Bonferroni's posttest).

As already reported by Cervellati et al. [33], 1 h exposure to GSM-217 Hz signals significantly increased Cx40 (175%; *P* < 0.001) and Cx43 (166%; *P* < 0.001) mRNA expression as compared to sham-exposed cells, whereas it did not change expression levels for Cx45 gene product (Figure 1(a)). 

Treatment with 10^−6 ^M 17-*β*-estradiol for 24 h significantly increased mRNA expression of Cx40 (178%; *P* < 0.001) and Cx43 (180%; *P* < 0.001) with respect to sham-exposed cells, whereas Cx45 mRNA remained unmodified (Figure 1). 

When cells pretreated with 10^−6 ^M 17-*β*-estradiol for 24 h were exposed to 1 h HF-EMF, we observed an increase for Cx40 and Cx43 (185%; *P* < 0.001, 189%; *P* < 0.001, resp.) mRNA expression similar to that found in the presence of HF-EMF or steroid hormone alone. As for Cx45 mRNA, also the combination of the two treatments did not induce any significant change (Figure 1(a)). 

All treatments produced no effect on protein expression, at the same experimental conditions (Figure 1(b)).

### 3.3. Effect of HF-EMF, 17-*β*-Estradiol, and Their Combination on Integrin Expression in HTR-8/SVneo Cells

**Figure 2:**
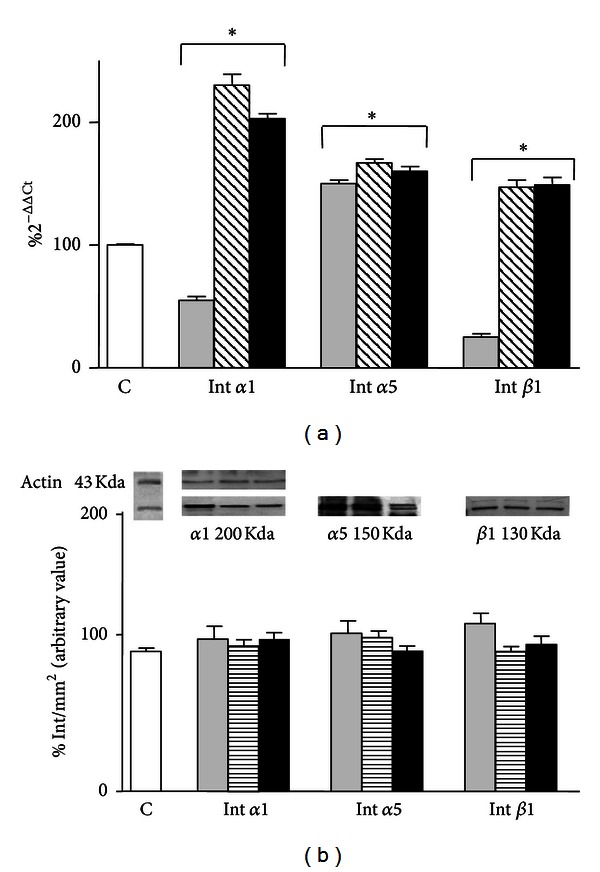
(a) Effect of HF-EMF (grey bar), 10^−6 ^M 17-*β*-estradiol (hatched bar), and their combination (black bar) on the expression of Int *α*1, Int *α*5, and Int *β*1 mRNA in HTR-8/SVneo cells. Results are expressed in %  2^−ΔΔCT^ with respect to the control value (white bar). (b) Western blot detection of Integrins Int *α*1, Int *α*5, and Int *β*1 subunit proteins expression in HTR-8/SVneo cells HF-EMF exposed. Results are the means ± SEM of three independent experiments, each analysed in triplicate. Data are means ± SEM of at least three independent experiments. *P < 0.001 versus sham-exposed cells (one-way ANOVA followed by Bonferroni's posttest).

HF-EMF exposure for 1 h significantly decreased *α*1 subunit (55%; *P* < 0.001) and *β*1 subunit (25%; *P* < 0.001) mRNA levels, but it significantly enhanced *α*5 subunit (+50%; *P* < 0.001) mRNA expression with respect to sham-exposed cells (Figure 2(a)). 

All the integrin subunit mRNA expressions were significantly increased (230%, *P* < 0.001 for *α*1; 167%, *P* < 0.001 for *α*5; 127%, *P* < 0.001 for *β*1) by 24 h treatment with 10^−6 ^M 17-*β*-estradiol (Figure 2(a)).

An effect similar to that obtained in the presence of 17-*β*-estradiol treatment was found when the cells were pretreated with the hormone for 24 h and then exposed to 1 h HF-EMF. In fact mRNA expression for all integrins tested significantly augmented with respect to sham-exposed cells (203%, *P* < 0.001 for *α*1; 160%, *P* < 0.001 for *α*5; 118%, *P* < 0.001 for *β*1) (Figure 2(a)).

All treatments produced no effect on protein expression, at the same experimental conditions (Figure 2(b)).

### 3.4. Expression of ER Subtypes in Human Chorionic Villi and HTR-8/SVneo Cells

**Figure 3:**
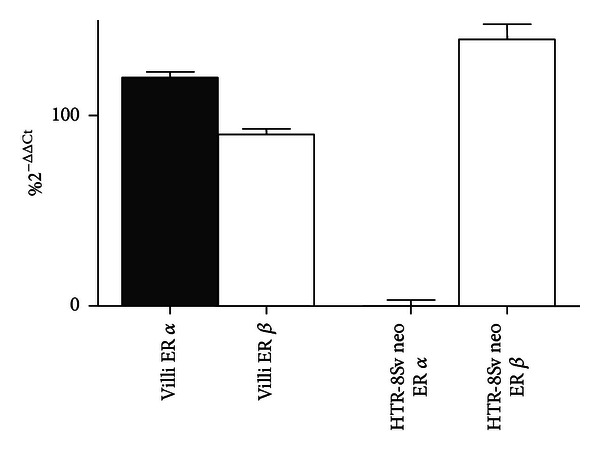
Expression of ER in first trimester human villi and in HTR-8/SVneo cells. Results are expressed in %  2^−ΔΔCT^ with respect to the housekeeping gene. Data are means ± SEM of at least three independent experiments. *P < 0.001 versus sham-exposed cells (one-way ANOVA followed by Bonferroni's posttest).

Both ER-*α* and ER-*β* subtypes were expressed in human chorionic villi, as previously reported [26]. In HTR-8/SVneo cells, instead, only ER-*β* isoform was present whereas ER-*α* isoform was not detectable (Figure 3).

### 3.5. Effect of HF-EMF, 17-*β*-Estradiol, and Their Combination on ER-*β* Expression in HTR-8/SVneo Cells

**Figure 4:**
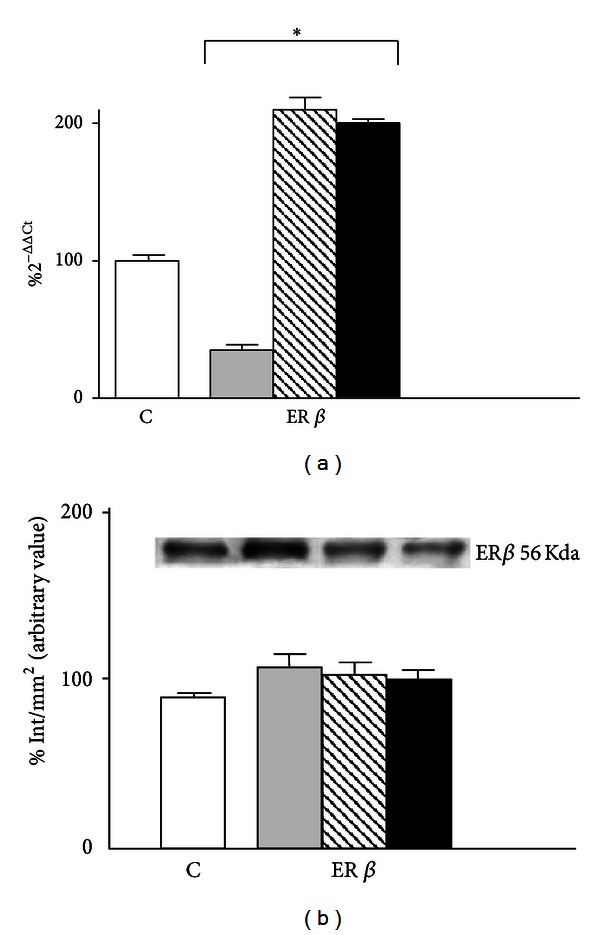
(a) Effect of HF-EMF (grey bar), 10^−6 ^M 17-*β*-estradiol (hatched bar), and their combination (black bar) on the expression of ER*β* mRNA in HTR-8/SVneo cells. Results are expressed in %  2^−ΔΔCT^ with respect to the control value (white bar). (b) Western blot detection of ER*β* protein expression in HF-EMF-exposed HTR-8/SVneo cells. Results are the means ± SEM of three independent experiments, each analysed in triplicate. Data are means ± SEM of at least three independent experiments. *P < 0.001 versus sham-exposed cells (one-way ANOVA followed by Bonferroni's posttest).

The 1 h exposure to GSM-217 Hz signal significantly reduced mRNA expression of ER-*β* (35%, *P* < 0.001) whereas 10^−6^ M 17-*β*-estradiol for 24 h significantly increased the receptor mRNA expression (210%, *P* < 0.001), with respect to sham-exposed cells. A stimulatory effect (180%, *P* < 0.001) on ER-*β* mRNA level was also found when cells pretreated with the steroid hormone for 24 h were then exposed for 1 h to HF-EMF (Figure 4(a)).

All treatments produced no effect on protein expression, at the same experimental conditions (Figure 4(b)).

### 3.6. Effect of HF-EMF, 17-*β*-Estradiol, and Their Combination on ER-*β*  Immunolocalization in HTR-8/SVneo Cells

**Figure 5:**
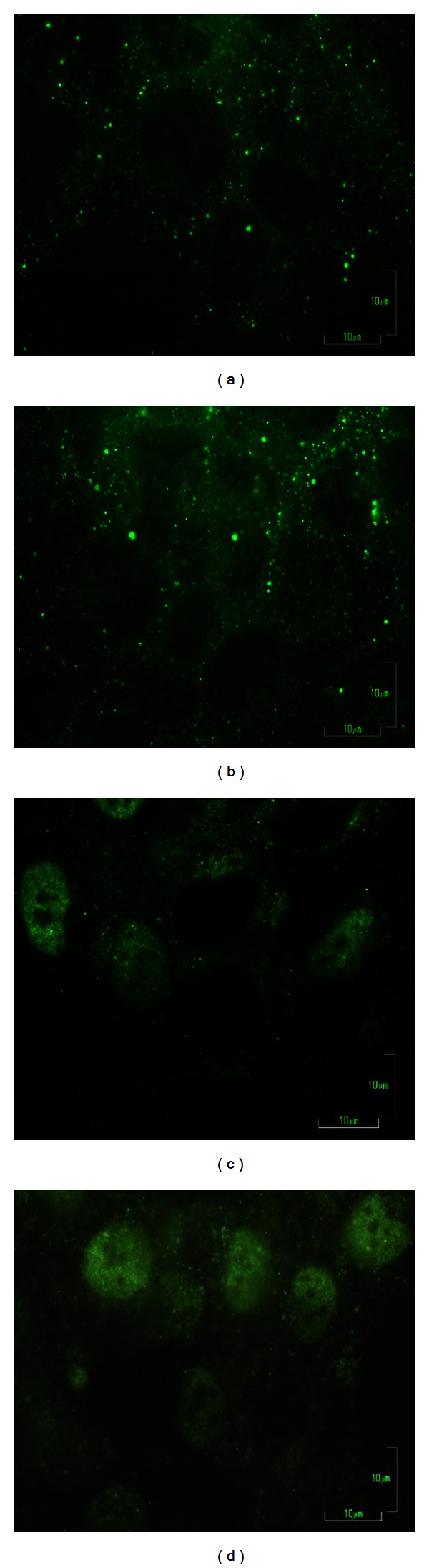
Indirect immunofluorescence staining of ER*β* in HTR-8/SVneo cells. (a) Sham-exposed cells; (b) HF-EMF-exposed cells; (c) sham-exposed + 10^−6 ^M 17-*β*-estradiol; (d) HF-EMF + 10^−6 ^M 17-*β*-estradiol exposed cells. Scale bars = 10 *μ*m.

In sham-exposed cells we found punctuate fluorescence for ER-*β* predominantly in the cytoplasm (Figure 5(a)). The 1 h HF-EMF irradiated cells showed a fluorescence distribution for ER-*β* comparable to that observed in sham-exposed cells (Figure 5(b)). When the cells were treated with 10^−6 ^M 17-*β*-estradiol for 24 h, a nuclear translocation of ER-*β* fluorescence was observed (Figure 5(c)). A similar effect was induced by pretreatment with 10^-6 ^M 17-*β*-estradiol for 24 h followed by 1 h exposure to HF-EMF (Figure 5(d)).

### 3.7. Effect of HF-EMF, 17-*β*-Estradiol, and Their Combination on Ultrastructural Features in HTR-8/SVneo Cells

**Figure 6:**
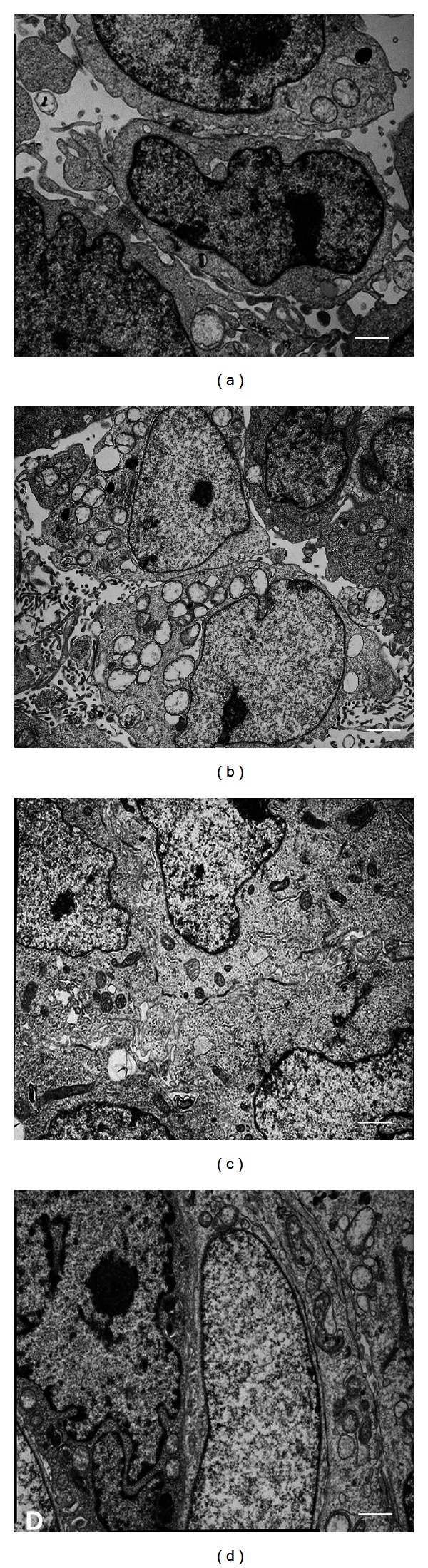
Electron microscopy of HTR-8/SVneo confluent culture cells under different experimental conditions. (a) Sham exposed-cells; (b) HF-EMF exposed cells; (c) 10^−6 ^M 17-*β*-estradiol; (d) 10^−6 ^M 17-*β*-estradiol + HF-EMF exposed cells. Bars = 2 *μ*m.

As already reported [33], electron microscopy examinations of selected areas of sham-exposed HTR-8/SVneo cells showed neighbouring cells in apposition with each other (Figure 6(a)). A decrease in cellular adhesion was found when cells were exposed to 1 h HF-EMF (Figure 6(b)). On the contrary, following 10^−6 ^M 17-*β*-estradiol treatment for 24 h, HTR-8/SVneo cells formed tightly adherent cellular islets (Figure 6(c)). Cells exposed for 1 h to HF-EMF and pretreated with the steroid hormone for 24 h did not show ultrastructural morphological changes in comparison with cells treated with 10^−6 ^M 17-*β*-estradiol alone (Figure 6(d)).

## 4. Discussion

Human placental development critically relies upon the differentiation of cytotrophoblast stem cells towards the villous and the invasive extravillous pathways. During this process a pivotal role is played by interactions between trophoblast cells and extracellular matrix, mediated by various kinds of adhesion molecules such as Cxs and Ints [16]. For instance, trophoblast cells lose *α*6*β*4 and gain *α*5*β*1 and *α*1*β*1 Ints [40], and they decrease Cx40-containing gap junctions, while increasing *α*1 integrin expression [13, 41]. Expressions of both Cxs and Ints are affected by hormones [15] as well as by environmental stresses [42, 43]. 

As for hormonal regulation of Cx expression, estrogens were reported to increase Cx43 and cell to cell communication in human myometrial cultured cells [44]. Moreover, they induce Cx26 and Cx43 in rat endometrium during preimplantation, implantation, and decidualization [45]. In human trophoblast 17-*β*-estradiol regulates the expression of Cx43 that is involved in differentiation from cyto- to syncytiotrophoblast, as well as EVT from the proliferative to the invasive phenotype [12]. In the present work, using a well-characterized model of human EVT, the HTR-8/SVneo cell line, we investigated the effect of HF-EMF, estradiol, and their combination on Cx, Int, and ER expression as well as on cell ultrastructure. In our cell line either HF-EMF or 17-*β*-estradiol, both alone or in combination, increased Cx40 and Cx43 mRNA expression leaving unaltered Cx45 transcript. As already showed in our foregoing paper [33] with regard to HF-EMF effect on Cx protein expression, in the present study 17-*β*-estradiol and its combination with HF-EMF did not induce any change in Cx protein levels. Accordingly, a discrepancy between mRNA transcript and protein expression had already been reported both in EVT cells and other cell types regarding different genes and proteins [46, 47]. 

As for Ints, the expressions of *α*1, *α*5, and *β*1 were already shown in HTR-8/SVneo cells [48, 49].

Very little is known, however, about the effect of HF-EMF on Int expression. Pulsed electromagnetic fields had no effect in osteosarcoma cell line [50], but extremely low frequency magnetic fields induced a segregation of *α*4 integrin in human keratinocytes, suggesting an interference with cellular adhesion [51]. In human decidua during early pregnancy the regulation of extracellular matrix remodeling as well as integrin switching is at least partially modulated by reproductive hormones [15, 52]. 

In our study we demonstrate, for the first time, that both HF-EM and 17-*β*-estradiol were able to modulate the expression of these adhesion molecules. In fact 1 h exposure to HF-EMF decreased *α*1 and *β*1 Int subunit mRNA levels, while increasing *α*5 transcript. On the contrary 17-*β*-estradiol induced an enhancement of all the Int subunit mRNA expressions. These data suggest that the hormone may exert an action promoting trophoblast differentiation along the invasive pathway, contrary to HF-EMF. Moreover, 17-*β*-estradiol effect seemed to prevail over the electromagnetic field one, since the treatment with both agents provoked results comparable to those obtained in the presence of the estrogen alone. However, once again, no effect on protein expression was detected in any experimental condition. Accordingly, 24 h estradiol treatment did not affect Int *α*5 and Int *β*4 proteins in human decidua [52]. Nevertheless, integrin protein expression enhancement by estradiol was reported in other experimental conditions [15, 53]. 

Conflicting results have been reported on estrogen receptor in human placenta [15, 26, 54], although the *α* isoform has been found in first trimester human chorionic villi [55].

However, in our study, we detected the presence of the ER-*β* isoform, while ER-*α* isoform was undetectable either in basal or in stimulated conditions, thus suggesting a mature state of differentiation for HTR-8/SVneo cells [26]. Therefore, 17-*β*-estradiol stimulatory action on both Cx and Int mRNA levels should be mediated by the ER-*β* isoform receptor. In our data ER-*β* receptor subtype mRNA was reduced by HF-EMF exposure but enhanced by 17-*β*-estradiol treatment. Also in this case the HF-EMF reductive effect was blinded by 24 h hormone pretreatment. These data suggest a putative autocrine action of estrogen on its own receptor in HTR-8/SVneo cells, as already reported in placental cells with regard to ER-*α* [15]. Although no significant modifications at the protein level were found, localization of ER-*β* isoform was notably influenced by hormonal treatment. In fact a nuclear translocation of ER-*β* fluorescence became evident after estrogen exposure, also in the presence of HF-EMF which, per se, did not alter the cytoplasmic localization.

Ultrastructural observation seemed to reflect the results found at the Int  mRNA level. In fact, hormone treatment ameliorated adhesion between neighboring cells, favoring the formation of compact cellular islet. HF-EMF exposure, instead, seemed to increase the distance between adjacent cells. Moreover, estradiol was able to preserve the ultrastructural features of HTR-8/SVneo cells also in the presence of the electromagnetic field. 

Thus it can be hypothesized that estradiol may facilitate decidual stroma invasion reinforcing adhesion between cells and extracellular matrix, probably through a modulation of Int subunits, even if we were not able to detect any effect on Int protein expression. This may be due to translational and posttranslational regulation of these adhesion molecules [56]. It is well established that antibodies that interfere with integrin ligand occupancy not only inhibit cell attachment to the ECM but also inhibit cell movement [57].

At this regard it is important to consider that a dysregulation of the previously mentioned molecules is associated to pregnancy disorders such as preeclampsia, IUGR, and preterm labour [16]. As for the mechanism of 17-*β*-estradiol action, the protective efficacy of the hormone against oxidative stress can be hypothesized. Indeed, in ARTE-19 cells it has been shown to exert an ER-*β*-mediated cytoprotection through the preservation of mitochondrial function, reduction of reactive oxygen species production, and induction of cellular antioxidant genes [58].

## 5. Conclusions

Growing attention is devoted today, even from international political institutions, to the influence of HF-EMF on human health, in particular following the recent report that exposure to cell phone radiofrequency signal increases brain glucose metabolism [59]. In the context of human pregnancy protection, it appears mandatory not only to investigate the effects of HF-EMF on implantation, morphogenesis, and fetal development, but also to ascertain the possible existence of protective physiological control mechanisms. At this regard, our study shows, for the first time, that 17-*β*-estradiol is able to counteract the effects of HF-EMF on trophoblastic Cx, integrins, and ER.

## Figures and Tables

**Table 1:** Primer sequences and PCR condition.

Gene	Primer sequence	*T*_*a*_ °C	Product length (bp)	QPCR amplification efficiency* (%)	No. of cycles	Reference primer bank
Cx40	F: 5′-tcctggaggaagtacacaagc-3′ R: 5′-atcacaccggaaatcagcctg-3′	60.1	137	97.2	39	GenBank Accession NM 181703

Cx43	F: 5′-tcaagcctactcaactgctgg-3′ R: 5′-tgttacaacgaaaggcagactg-3′	60.4	125	98.4	39	GenBank Accession NM 000165

Cx45	F: 5′-atgagttggagctttctgactcg-3′ R: 5′-cggctgttctgtgttgcac-3′	60.4	174	94.5	39	GenBank Accession NM 005497

Int *α*1	F: 5′-tgctgctggctcctcactgttgtt-3′ R: 5′-gggcccacaagccagaaatcct-3′	60.6	354	95.8	39	GenBank Accession NM 181501.1

Int *α*5	F: 5′-gaaccagagccgcctgctgg-3′ R: 5′-gagcctccacggagagccga-3′	60.8	215	95.8	39	GenBank Accession NM 002205.2

Int *β*1	F: 5′-acgccgcgcggaaaagatgaatt-3′ R: 5′-acccacaatttggccctgcttg-3′	60.5	155	95.4	39	GenBank Accession NM 002211.3

RPL13A	F: 5′-cctaagatgagcgcaagttgaa-3′ R: 5′-ccacaggactagaacacctgctaa-3′	60.2	203	97.3	39	Pattyn et al. 2006 [34]

RPL11A	F: 5′-tgcgggaacttcgcatccgc-3′ R: 5′-gggtctgccctgtgagctgc-3′	60.1	108	96.5	39	GenBank Accession NM 000975.2

GAPDH	F: 5′-tgacgctggggctggcattg-3′ R: 5′-ggctggtggtccaggggtct-3′	60	134	94.6	39	GenBank Accession NM 002046.3

Data calculated by OpticonMonitor 3 Software (Bio-Rad).
